# Immunity to Visceral Leishmaniasis

**DOI:** 10.1155/2012/780809

**Published:** 2011-12-28

**Authors:** Nahid Ali, Asrat Hailu Mekuria, Jose M. Requena, Christian Engwerda

**Affiliations:** ^1^Infectious Diseases and Immunology Division, Indian Institute of Chemical Biology, 4 Raja, S. C. Mullick Road, Kolkata-32, West Bengal Kolkata 700032, India; ^2^Faculty of Medicine, Addis Ababa University, P. O. Box 28017-1000, Addis Ababa, Ethiopia; ^3^Departamento de Biología Molecular Centro de Biología Molecular “Severo Ochoa” C/ Nicolás Cabrera, 1 Universidad Autónoma de Madrid, 28049 Madrid, Spain; ^4^Australian Centre for Vaccine Development, Queensland Institute of Medical Research, 300 Herston Road, Herston, QLD 4006, Australia

Leishmaniasis is a major vector-borne parasitic disease affecting 12 million people worldwide. With a broad range of clinical manifestations, ranging from self-healing skin ulcers to disfiguring mucosal lesions to life-threatening infections of visceral organs (liver and spleen), the disease has become a serious human health issue, particularly in developing countries. Among all of its forms, visceral leishmaniasis (VL, also known as kala-azar), caused by the *Leishmania donovani* complex (i.e., *L. donovani* and *L. infantum* in Old World and *L. chagasi* in New World), is often fatal in the absence of treatment. Although humans are the principal hosts for *L. donovani*, canine species are the main reservoirs of *L. infantum*. Canine VL affects millions of dogs and is associated with outbreaks of human VL and hence has become a major public health issue. The lack of vaccines to prevent and/or treat these infections, as well as the emergence of drug resistant parasites, is serious impediments to control leishmaniasis. Therefore, developing new prophylactic and therapeutic strategies against this disease is urgently required. However, for this to occur, a better understanding of the complex immune mechanisms generated in response to infection and defining those involved in resistance to infection is required. In this special issue on “*Immunity to visceral leishmaniasis*”, several selected papers will discuss these issues. 

The first paper in this special issue describes recent advances in vaccination strategies against VL. To date there is no vaccine available for any form of human leishmaniasis. The lack of knowledge relating to disease pathogenesis, the rise of coinfection with HIV, and cost of development are significant hindrances to vaccine development. Therefore, to reduce the burden of VL, particularly in resource poor nations, increased investment in VL vaccine development is required. The second paper in this issue focuses on the utility of live-attenuated parasites as vaccine candidates. Although subunit *Leishmania* vaccines have shown some efficacy in animal models, they have not yet been shown to have protective effects in humans. The recuperation of infected individuals and subsequent protection from reinfection indicates that infection with the parasite may be a prerequisite for the development of protective immunity. Thus, to achieve long lasting protection, genetically altered live-attenuated parasites could be a valuable tool. The third paper in this issue discusses recent advances made in cytokine and phenotypic cell profiles in different tissues and organs of dogs infected with *L. infantum*. Dogs represent an important reservoir of parasites in some regions, and infected dogs have been linked with human VL cases. This paper describes some of the complex immune responses developed by the dog in response to infection. The fourth paper in the issue describes evasion mechanisms employed by parasites that enable them to persist within the host. Parasites modulate the host immune response in the spleen and liver differently in mouse models, as indicated by a distinct pattern of organ-specific parasite growth during disease progression. While infection in the liver resolves within 6–8 weeks, a chronic infection becomes established in the spleen. Although the exact immune mechanisms responsible for parasite persistence in spleen are not known, it is now clear that the parasite plays an important role in this process. The fifth paper in the issue describes novel immune evasion mechanisms employed by the *Leishmania* parasite, and in particularly, those used by virulent promastigote and amastigote forms that overexpress a glycoprotein enzyme (ectonucleotidase) on the parasite plasma membrane. The ectonculeotidase, with its catalytic sites facing extracellularly, hydrolyses extracellular nucleotides resulting in the production of adenosine. Increased levels of adenosine aid in the establishment of infection. Thus, the discovery of the immunomodulatory effects of adenosine and the characterisation of ectonucleotidases provide new insights into complex immune responses during leishmaniasis. The final paper in this issue discusses *Leishmania*-mediated manipulation of the signalling machinery of the host cell that enables parasite persistence. The identification of the key signalling mechanisms modulated by parasites will help to understand the strategies used by parasites to subvert the host immune system. Moreover, given that these signalling pathways could be manipulated pharmacologically, an improved understanding of the host-parasite interaction may allow the development of new therapies to control leishmaniasis.

*Nahid Ali* *Nahid Ali* *Asrat Hailu Mekuria* *Asrat Hailu Mekuria* *Jose M. Requena* *Jose M. Requena* *Christian Engwerda* *Christian Engwerda*

